# Plasma symmetric dimethylarginine as a metabolite biomarker of severe acute ischemic stroke

**DOI:** 10.3389/fneur.2024.1472424

**Published:** 2024-11-07

**Authors:** Saana Pihlasviita, Olli S. Mattila, Tiina Nukarinen, Markku Kuisma, Heini Harve-Rytsälä, Juhani Ritvonen, Gerli Sibolt, Sami Curtze, Daniel Strbian, Mikko Pystynen, Turgut Tatlisumak, Perttu J. Lindsberg

**Affiliations:** ^1^Neurology and Clinical Neurosciences, University of Helsinki and Helsinki University Hospital, Helsinki, Finland; ^2^Emergency Medicine and Services, Department of Emergency Care, University of Helsinki and Helsinki University Hospital, Helsinki, Finland; ^3^Department of Clinical Neuroscience/Neurology, Institute of Neuroscience and Physiology, Sahlgrenska Academy at University of Gothenburg, Gothenburg, Sweden; ^4^Department of Neurology, Sahlgrenska University Hospital, Gothenburg, Sweden

**Keywords:** stroke, diagnosis, acute management, biomarkers, SDMA

## Abstract

**Introduction:**

After severe ischemic stroke (IS), circulating levels of symmetric dimethylarginine (SDMA) increase. We investigated the early dynamics of SDMA in stroke to potentially aid with prehospital identification of severe IS from hemorrhagic stroke (HS).

**Methods:**

We performed targeted mass spectrometry (MS) measurements of SDMA in two sequential acute plasma samples (early and secondary) of 50 IS patients with LVO and 49 HS patients. Secondary samples of 227 IS and 84 HS patients with moderate to severe symptoms (NIHSS ≥ 7) subsequently underwent ELISA validation.

**Results:**

The median (IQR) last-known-well (LKW) to sampling times were 43 min (35–67) for early samples in the MS analysis, and 83 min (65–113) for secondary samples in MS and ELISA analyses. No inter-group differences existed in early samples, but IS patients had significantly higher mean (IQR) SDMA levels in secondary samples in both analyses: 5.8 (5.3–6.9) vs. 5.1 (4.2–5.8) A.U. for HS, *p* < 0.001, with MS; and 0.82 (0.72–1.01) vs. 0.71 (0.58–0.85) nmol/mL for HS, *p* < 0.001, with ELISA. For IS patients, higher SDMA levels were associated with cardioembolic stroke: 0.84 (0.73–1.09) vs. 0.79 (0.71–0.91) nmol/mL for other etiologies, *p* = 0.042, and poor outcome: modified Rankin Scale (mRS) 4–6; 0.90 (0.73–1.06) vs. 0.80 (0.72–0.97) nmol/mL for mRS 0–3 (*p* = 0.045).

**Conclusion:**

In a large clinical cohort of stroke patients with moderate to severe symptoms, our data suggest that SDMA can assist in differentiation of IS and HS patients already 1 h and a half after symptom onset. SDMA may prove to have future value in a diagnostic stroke biomarker panel.

## 1 Introduction

The prehospital management of both ischemic and hemorrhagic stroke (IS and HS) is highly time-dependent and benefit from early differential diagnosis to provide optimal prehospital care and direct transfer to an adequately equipped hospital for immediate therapy. In most hospital districts, because deployment of high-cost mobile stroke units has been limited to metropolitan areas, emergency medical services (EMS) lack affordable diagnostic methods for differentiating IS from HS. The recently published INTERACT 4 trial (1) reported divergent effect of prehospital blood-pressure reduction in IS and HS patients, indicating that ideal prehospital management differs greatly between stroke subtypes. The routine use of mechanical thrombectomy in IS patients with large vessel occlusion (LVO) has further emphasized the importance of precision in early triage. Prehospital recognition of LVO would enable timely preparation of an angiography suite prior to hospital arrival, but clinical prehospital LVO scales have shown limited performance in ruling out HS (2). Thus, novel diagnostic methods are needed to optimize prehospital care, to improve triage, and to select patients for future prehospital therapeutic studies.

One promising biomarker candidate for ischemic stroke is the proteolytic metabolite symmetric dimethylarginine (SDMA). This is produced in all nucleated cells during proteolysis when methylated arginine residues of proteins are released into the cytosol (3), with preclinical studies finding the highest tissue concentrations in the brain (4). Upon intracellular accumulation, SDMA is readily liberated into the extracellular space and systemic circulation and can be measured in plasma as a circulating metabolite. SDMA, eliminated mainly via renal excretion, can also serve as a marker for renal function (5).

Preliminary studies have proposed that, in the acute phase of IS, circulating levels of SDMA rise within 6 h of symptom onset (6), and may associate with cardioembolic (CE) stroke etiology (7, 8). In contrast, SDMA levels have not been found to significantly increase in the acute phase of HS (8, 9). Further, elevated levels of SDMA following IS have predicted poor outcome as well as all-cause mortality (10–12). Elevated circulating concentrations of SDMA have also been associated across differing patient populations with cardiovascular disease and all-cause mortality (13). Because circulating levels of SDMA elevate in acute IS, SDMA may have potential as a metabolite biomarker, either on its own or within a biomarker panel, in early differential diagnosis and prognostication of acute stroke patients. Because most diagnostic stroke biomarkers are released by HS (14, 15), those elevated in IS are rarer and of great value. We thus set out to define the very early dynamics of plasma SDMA in acute stroke and its usefulness as a biomarker for early differentiation of IS from HS patients, with a focus on severe stroke cases, the patient group requiring most urgent optimal treatment and transport decisions.

## 2 Methods

### 2.1 Study design

The Helsinki Ultra-Acute Stroke Biomarker Study is an observational project aiming to improve diagnostics of acute stroke through discovery of novel blood biomarkers (16, 17). In the hospital district of Helsinki and Uusimaa, all patients considered to be candidates for stroke recanalization therapies, known as stroke code (SC) patients, are transported to the emergency department of Helsinki University Hospital (HUH), the district's only 24/7 neurological service. Emergency medical services (EMS) of the region operate under central management, and all units are staffed by professional emergency medicine technicians or paramedics or both. Prehospital identification of stroke symptoms is based on the Face Arm Speech Test (FAST), and a phone consultation with an on-call neurologist or EMS physician is always available. Contrary to the practice of most other stroke biomarker studies using in-hospital sampling, we trained our EMS personnel to collect prehospital blood samples on site from all SC patients before transportation (17).

**Figure 1:**
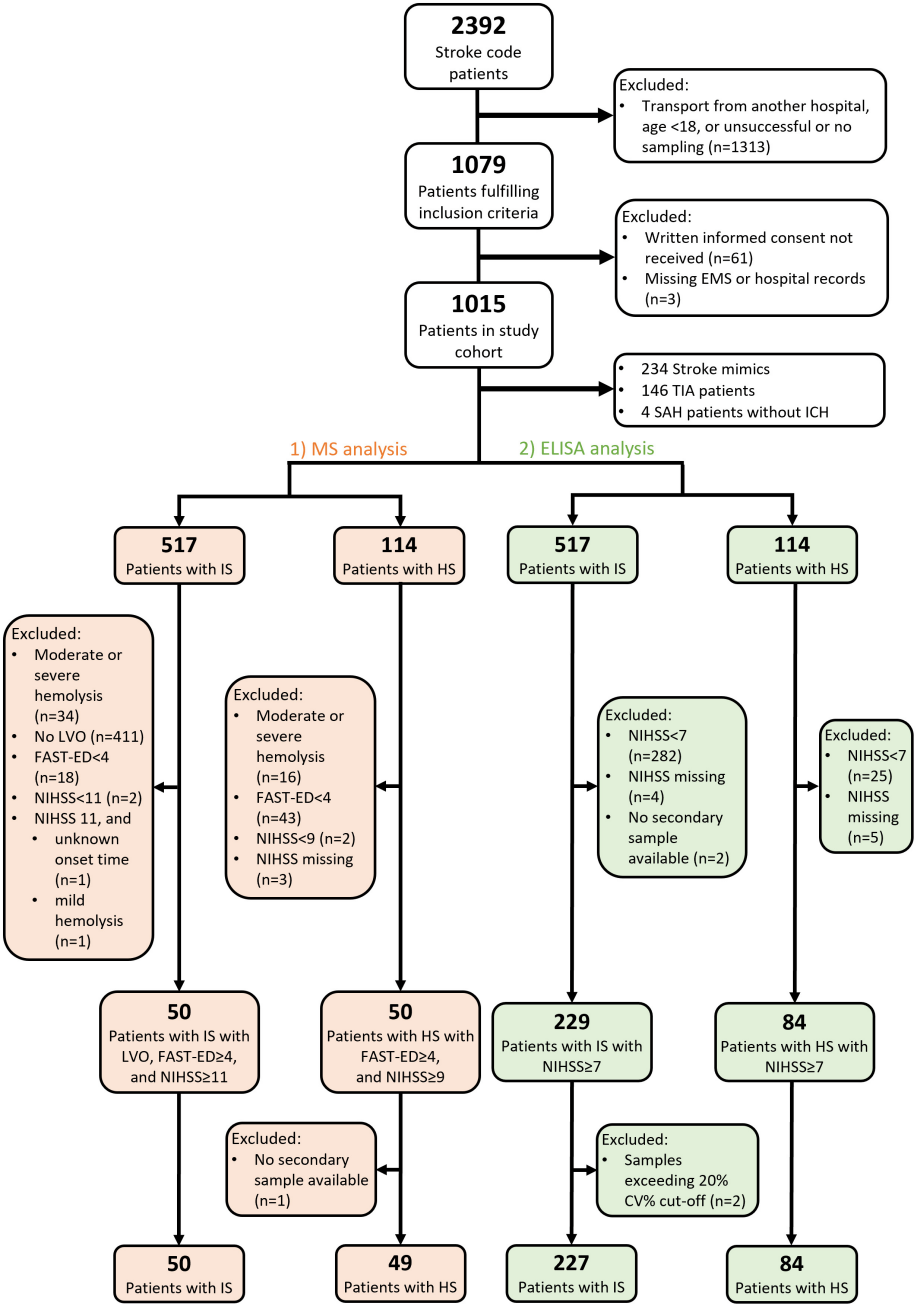
Flowchart of patient selection for MS and ELISA analyses. EMS, emergency medical services; TIA, transient ischemic attack; SAH, subarachnoid hemorrhage; ICH, intracranial hemorrhage; IS, ischemic stroke; HS, hemorrhagic stroke; LVO, large vessel occlusion; FAST-ED, Field Assessment Stroke Triage for Emergency Destination; NIHSS, National Institutes of Health Stroke Scale; CV, coefficient of variation.

Of the 2,392 SC patients admitted to HUH during the study enrolment period between May 20, 2013, and November 19, 2015, 1,015 were included in the final Helsinki Ultra-Acute Stroke Biomarker Study cohort (Figure 1) (16). Following completion of all follow-up investigations, chart review allowed data collection into the study database (16). The National Institutes of Health Stroke Scale (NIHSS) was recorded upon admission, and previously described cut-off limits of moderate and severe strokes (NIHSS > 8 and NIHSS > 15, respectively) served for univariate and area under the receiver-operating characteristics curve (AUC) analyses (18). The definition of poor outcome was 3-month modified Rankin Scale (mRS) score 4–6 (19). The final diagnosis group (IS vs. HS) was based on all available patient records (16). The Field Assessment Stroke Triage for Emergency Destination (FAST-ED) scores to detect LVO (20) were counted retrospectively based on recorded admission NIHSS evaluations, in our district, gaze palsy and neglect not being routinely evaluated by EMS.

We analyzed the diagnostic performance of plasma SDMA in two phases with two complimentary measuring techniques (Figure 1). First, to compare prototypical cases, we selected a representative screening sample of 100 cases with either severe ischemic or hemorrhagic stroke and performed targeted measurements of SDMA in a mass spectrometry (MS) analysis in two sequential acute samples: early samples collected in the prehospital setting and secondary samples collected on hospital admission. To select patients with the most severe strokes in the MS analysis, we only included patients with a positive LVO screening score (FAST-ED ≥ 4) (21) and excluded IS patients without a verified LVO on admission CT-angiography (ICA or M1 occlusion). Of the remaining IS and HS patients, we selected 50 cases with the highest NIHSS scores in each diagnosis group.

In the second phase of the study, we aimed to further validate the differential diagnostic performance of SDMA in an ELISA analysis of a larger series of 227 IS and 84 HS patients, including 98 patients from the MS analysis. We used a threshold of NIHSS ≥ 7, a previously described optimal cut-off value of total NIHSS score to predict LVO (22).

### 2.2 Sample collection and storage

We have described the EMS training and prehospital sample collection (16, 17). Briefly, EMS personnel collected prehospital serum and plasma samples immediately after the on-scene identification of a SC patient by using a cannula adapter and vacuum tubes. In addition to primary prehospital samples, secondary acute samples were collected immediately on hospital arrival. Once in the hospital laboratory, samples were centrifuged at 2,000 g for 10 min at 20°C and divided into cryotubes for storage at −80°C.

### 2.3 Targeted mass spectrometry analysis of plasma metabolites

In the first phase of the study, the Metabolomics Unit of the Institute for Molecular Medicine Finland (FIMM) performed targeted measurement of plasma metabolites, including SDMA by means of multiple reaction monitoring (MRM) on a ACQUITY UPLC-MS/MS system (Waters Corporation, Milford, MA). Overall, 100 metabolites were measured in a targeted manner, of which SDMA was analyzed for this sub-study. The metabolic profiling protocol has been published (23).

The targeted MS analyses occurred in two separate runs, first for early samples collected in the prehospital setting, then for subsequent secondary samples collected on admission from the same patients. To ensure the comparability of the measurements performed in separate assay runs, 10 samples from the first run were re-analyzed in the second run. We calculated the median percent change of SDMA for the ten control samples between the two runs. Final measurement values of the study samples from the second run were then corrected using the median percent change derived from these control samples [corrected concentration = 2nd run value × (1 + median percent change in 134 controls/100)].

One HS patient without an available secondary sample was excluded from all analyses. Although mass spectrometry measurements were performed with a standard curve for quantitation, due to the screening nature of the assay, we could not verify the absolute level of quantitation, and thus reported results as arbitrary units (A.U.).

### 2.4 ELISA

For the second phase of the study, we used a commercially available human SDMA ELISA kit from DLD Diagnostika GmbH which is based on acetylation of sample SDMA. Measurements took place in the Neurology Research Unit at the University of Helsinki according to kit inserts provided by the manufacturer. One sample with SDMA measurement above the highest point on the standard curve was re-analyzed at a 5-fold dilution. Inter-assay and intra-assay coefficients of variation (CV%) were 14.9% and 6.1%, respectively. Each sample was measured in duplicate. We determined a 20% CV% cut-off for variation between each measured duplicate. Samples with duplicates exceeding this limit were reanalyzed, and samples from two patients still exceeding this CV% limit after replication were excluded.

### 2.5 Statistical analysis

Continuous variables are presented as medians and interquartile ranges, and categorical variables as absolute counts and percentages. Univariate analyses utilized the Mann-Whitney U, χ2, or Fisher exact test where appropriate. The non-parametric Spearman rank test served for measuring correlation. Significance we set at *p* ≤ 0.05. We used univariate analyses to analyze intergroup differences of SDMA levels, and linear regression models to correct for possible confounding factors. Due to SDMAs strong elevation in renal impairment, (5) univariate and correlation analyses were repeated for patients with normal creatinine levels. We performed area under the receiver-operating characteristics curve (AUC) analyses to evaluate how well plasma SDMA levels differentiate IS cases from HS. AUCs of 0.9–1.0 have been considered excellent, 0.8–0.9 good, 0.7–0.8 fair, and < 0.7 poor. (24) AUC analysis and plotting served to determine optimal cut-off values, and cross-tabulations to calculate diagnostic measures. We performed all analyses with SPSS (v.25, IBM).

## 3 Results

This study utilized two complimentary measuring techniques: MS and ELISA analyses. We included 99 patients (50 with IS, 49 with HS) in the initial targeted MS analysis and 311 patients (227 with IS, 84 with HS) in the larger ELISA analysis (Figure 1). Ninety eight samples analyzed in both MS and ELISA analyses showed a significant correlation (ρ = 0.605, *p* < 0.001). For baseline characteristics (Table 1), no statistically significant differences emerged between IS and HS patients in the MS cohort, but in the ELISA cohort, IS patients had higher rates of underlying coronary artery disease (CAD; *p* = 0.016), of myocardial infarction (MI; *p* = 0.032), and of elevated creatinine (*p* = 0.019). HS patients presented with more severe symptoms, with a median admission NIHSS (IQR) of 15 (11–19) compared to 12 (9–17) in the IS group (*p* = 0.002).

**Table 1:** Univariate comparisons of baseline characteristics between IS and HS patients in MS and ELISA analyses.

**Variable**	**MS analysis**			**ELISA analysis**		
	**IS (*n =* 50)**	**HS (*n =* 49)**	***p*-Value**	**IS (*n =* 227)**	**HS (*n =* 84)**	***p*-Value**
Male, *n* (%)	29 (58.0)	35 (71.4)	0.162	120 (52.9)	54 (64.3)	0.072
Age, years, median (IQR)	65 (58–71)	67 (59–74)	0.493	71 (63–81)	69 (60–77)	0.075
Time from LKW to secondary sample, min, median (IQR)	77 (65–102)	93 (65–119)^a^	0.182	87 (65–133)^b^	91 (65–116)^c^	0.925
NIHSS on admission, median (IQR)	17 (14–20)	16 (13–20)	0.611	12 (9–17)	15 (11-19)	**0.002**
Creatinine level above reference range, *n* (%)	9 (18.0)	5 (10.2)	0.388	52 (22.9)^d^	9 (10.7)^e^	**0.019**
Hypertension, *n* (%)	25 (50.0)	33 (67.3)	0.103	144 (63.4)	59 (70.2)	0.286
Diabetes, *n* (%)	5 (10.0)	7 (14.3)	0.554	34 (15.0)	10 (11.9)	0.584
Hyperlipidemia, *n* (%)	16 (32.0)	16 (32.7)	1.000	100 (44.1)	28 (33.3)	0.093
Previous IS, *n* (%)	5 (10.0)	6 (12.2)	0.760	36 (15.9)	7 (8.3)	0.098
Atrial fibrillation, *n* (%)	12 (24.0)	7 (14.3)	0.308	59 (26.0)	15 (17.9)	0.177
Congestive heart failure, *n* (%)	4 (8.0)	3 (6.1)	1.000	22 (9.7)	5 (6.0)	0.369
Coronary artery disease, *n* (%)	7 (14.0)	4 (8.2)	0.525	46 (20.3)	7 (8.3)	**0.016**
Previous myocardial infarction, *n* (%)	4 (8.0)	2 (4.1)	0.678	23 (10.1)	2 (2.4)	**0.032**
Current smoking, *n* (%)	19 (38.0)	12 (24.5)	0.194	64 (28.2)	21 (25.0)	0.668

IS, ischemic stroke; HS, hemorrhagic stroke; MS, mass spectrometry; IQR, interquartile range; LKW, last known well; NIHSS, National Institutes of Health Stroke Scale. ^a^*n* = 46; ^b^*n* = 225; ^c^*n* = 81; ^d^*n* = 226; ^e^*n* = 83. Bold indicates *p* < 0.05.

### 3.1 MS analysis

The median (IQR) delay from last-known-well (LKW) to sampling was 43 min (35–67) for early samples, and 83 min (65–113) for secondary samples. We found no statistically significant difference in plasma SDMA between IS and HS groups in early samples (unadjusted *p* = 0.826). However, in secondary samples, IS patients had significantly higher plasma SDMA levels: 5.8 (5.3–6.9) vs. 5.1 (4.2–5.8) A.U. in HS (unadjusted *p* < 0.001). Notably, we found a significant correlation between plasma SDMA levels and stroke severity (NIHSS) on admission in IS patients (ρ 0.546, *p* < 0.001), but not in HS patients (ρ −0.184, *p* = 0.204). Correspondingly, the inter-group difference was more distinct in patients with severe stroke (NIHSS > 15), being 6.0 (5.6–7.6) for IS vs. 4.9 (4.1–5.3) A.U. for HS (unadjusted *p* < 0.001).

When comparing SDMA levels at two time points, no significant differences emerged in the rate of change between IS and HS groups: 0.13 (0.08–0.20) for IS vs. 0.12 (0.07–0.15) nmol/mL/min for HS group (*p* = 0.123, *n* = 96). However, rate of change was significantly correlated with admission NIHSS score in IS patients (ρ = 0.337, *p* = 0.017, *n* = 50), but not in HS patients (ρ = −0.015, *p* = 0.919, *n* = 46). Furthermore, there was no significant correlation with hemorrhage volume in HS patients (ρ = −0.225, *p* = 0.132, *n* = 98).

### 3.2 ELISA

**Figure 2:**
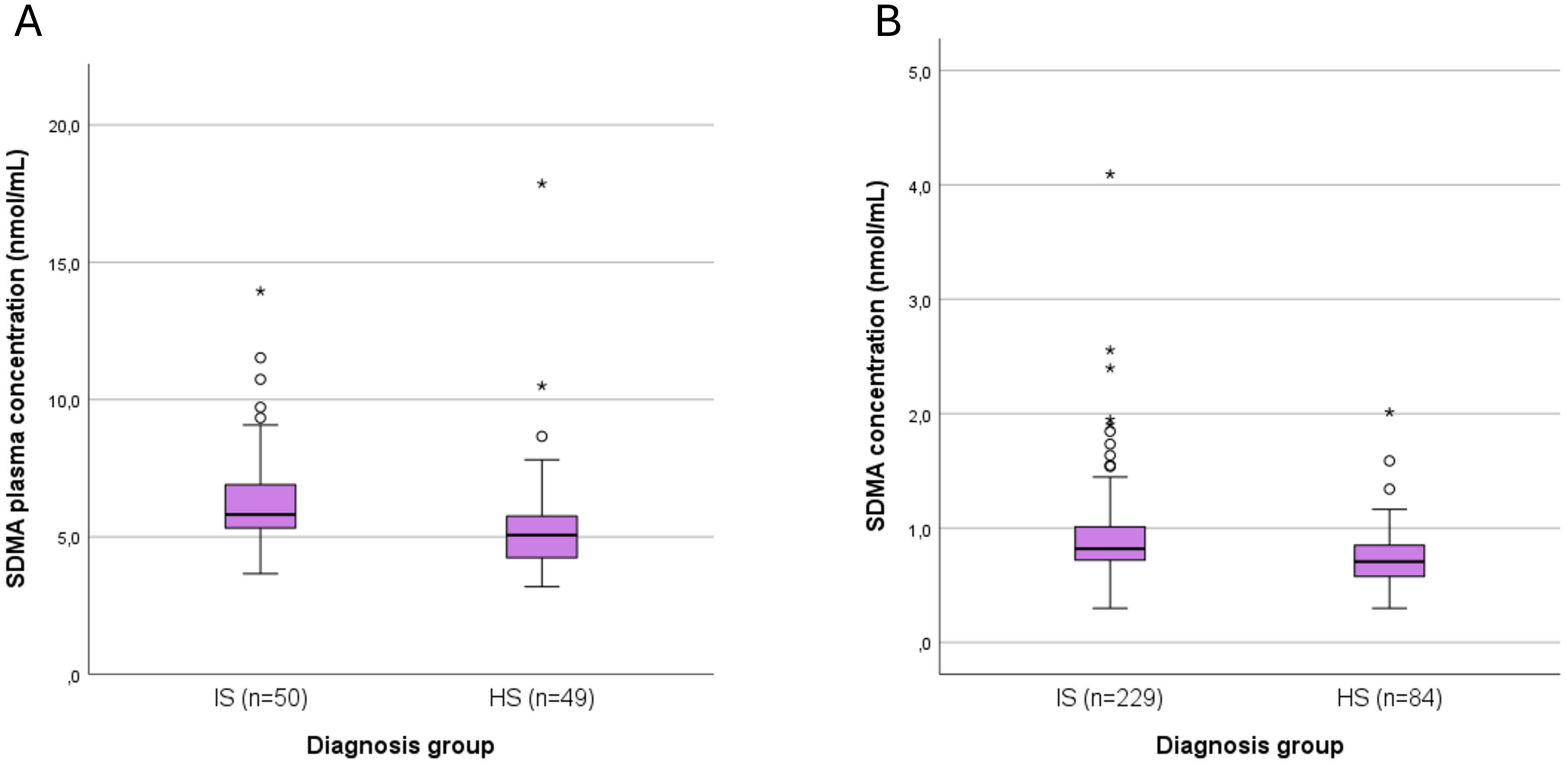
Box plots of plasma SDMA levels in the secondary samples of IS and HS patients in both **(A)** MS, and **(B)** ELISA analyses. SDMA, symmetric dimethylarginine; IS, ischemic stroke; HS, hemorrhagic stroke. Outliers are shown as small circles (>1.5 SD from mean) and stars (>3 SD from mean).

The delay from LKW to secondary sampling was 89 (65–125) min (*n* = 306). Again, plasma SDMA levels were significantly higher in IS patients: 0.82 (0.72–1.01) vs. 0.71 (0.58–0.85) nmol/mL for HS, unadjusted (*p* < 0.001). Box plots of plasma SDMA levels in the secondary samples of IS and HS patients in both MS and ELISA analyses are shown in Figure 2.

### 3.3 Regression analysis

To correct for possible confounding factors, we assessed results using linear regression in basic (age and sex) and full models (age, sex, creatinine level, NIHSS, CAD, and MI), as in Table 2. Diagnosis group (IS vs. HS) was a significant predictor of SDMA levels in the admission phase in both MS and ELISA models but not in the early phase in MS models. Of note, in the MS models, small sample size limited model stability; in three models we observed a non-normality of residuals, and in one model we observed heteroscedasticity (Table 2). This placed more importance on the results of our ELISA cohort, which showed neither of these imbalances.

**Table 2:** Linear regression models for early and secondary SDMA plasma levels in MS analysis, and for secondary SDMA plasma levels in ELISA analysis.

	**MS analysis**					
	**Early samples**			**Secondary samples**		
	**Model 1 (*****n** =* **99)**			**Model 1 (*****n** =* **99)**		
**Variable**	**B (CI 95%)**	**SE B**	***p*** **-Value**	**B (CI 95%)**	**SE B**	***p*** **-Value**
Age	0.003 (−0.007, 0.014)	0.005	0.525	0.050 (0.008–0.092)	0.021	**0.020**
Sex	0.033 (−0.203, 0.270)	0.119	0.781	−0.323 (-1.261, 0.616)	0.473	0.496
Diagnosis group	0.043 (−0.175, 0.260)	0.109	0.698	−1.046 (-1.908, −0.185)	0.434	**0.018**
**Variable**	**Model 2 (*****n** =* **99)**			**Model 2 (*****n** =* **99)**		
	**B (CI 95%)**	**SE B**	**p-Value**	**B (CI 95%)**	**SE B**	**p-Value**
Age	0.003 (−0.008, 0.014)	0.005	0.554	0.035 (0.008, 0.061)	0.013	**0.011**
Sex	0.055 (−0.199, 0.309)	0.128	0.670	0.665 (0.044, 1.287)	0.313	**0.036**
Diagnosis group	0.048 (−0.176, 0.272)	0.113	0.672	−0.700 (−1.249, −0.152)	0.276	**0.013**
NIHSS on admission	−0.003 (−0.030, 0.024)	0.013	0.815	0.042 (−0.024, 0.107)	0.033	0.207
Creatinine on admission	0.001 (−0.003, 0.005)	0.002	0.634	0.059 (0.049, 0069)	0.005	**< 0.001**
Coronary artery disease	−0.043 (−0.553, 0.467)	0.257	0.867	−1.145 (−2.393, 0.103)	0.628	0.072
Previous myocardial infarction	0.024 (−0.642, 0.690)	0.335	0.943	0.329 (−1.300, 1.958)	0.820	0.689
	**ELISA analysis**		
	**Secondary samples**		
	**Model 1 (*****n** =* **311)**		
**Variable**	**B (CI 95%)**	**SE B**	***p-*** **Value**
Age	0.008 (0.005–0.011)	0.289	**< 0.001**
Sex	−0.087 (−0.165, −0.010)	−0.124	**0.028**
Diagnosis group	−0.148 (−0.232, −0.065)	−0.188	**0.001**
	**Model 2 (*****n** =* **309)**		
**Variable**	**B (CI 95%)**	**SE B**	***p*** **-Value**
Age	0.003 (0.0004, 0.005)	0.102	**0.024**
Sex	0.068 (0.006, 0.131)	0.097	**0.032**
Diagnosis group	−0.089 (−0.154, −0.023)	−0.113	**0.008**
NIHSS on admission	4.29E-5 (−0.005, 0.005)	0.001	0.988
Creatinine on admission^a^	0.007 (0.006, 0.008)	0.675	**< 0.001**
Coronary artery disease	−0.005 (−0.105, 0.095)	−0.006	0.916
Previous myocardial infarction	−0.023 (−0.159, 0.114)	−0.018	0.746

SDMA, symmetric dimethylarginine; B, beta coefficient; CI, confidence interval; SE, standard error; NIHSS, National Institutes of Health Stroke Scale. ^a^*n* = 309. Bold indicates *p* < 0.05.

### 3.4 Analyses of patients with normal creatinine levels

**Figure 3:**
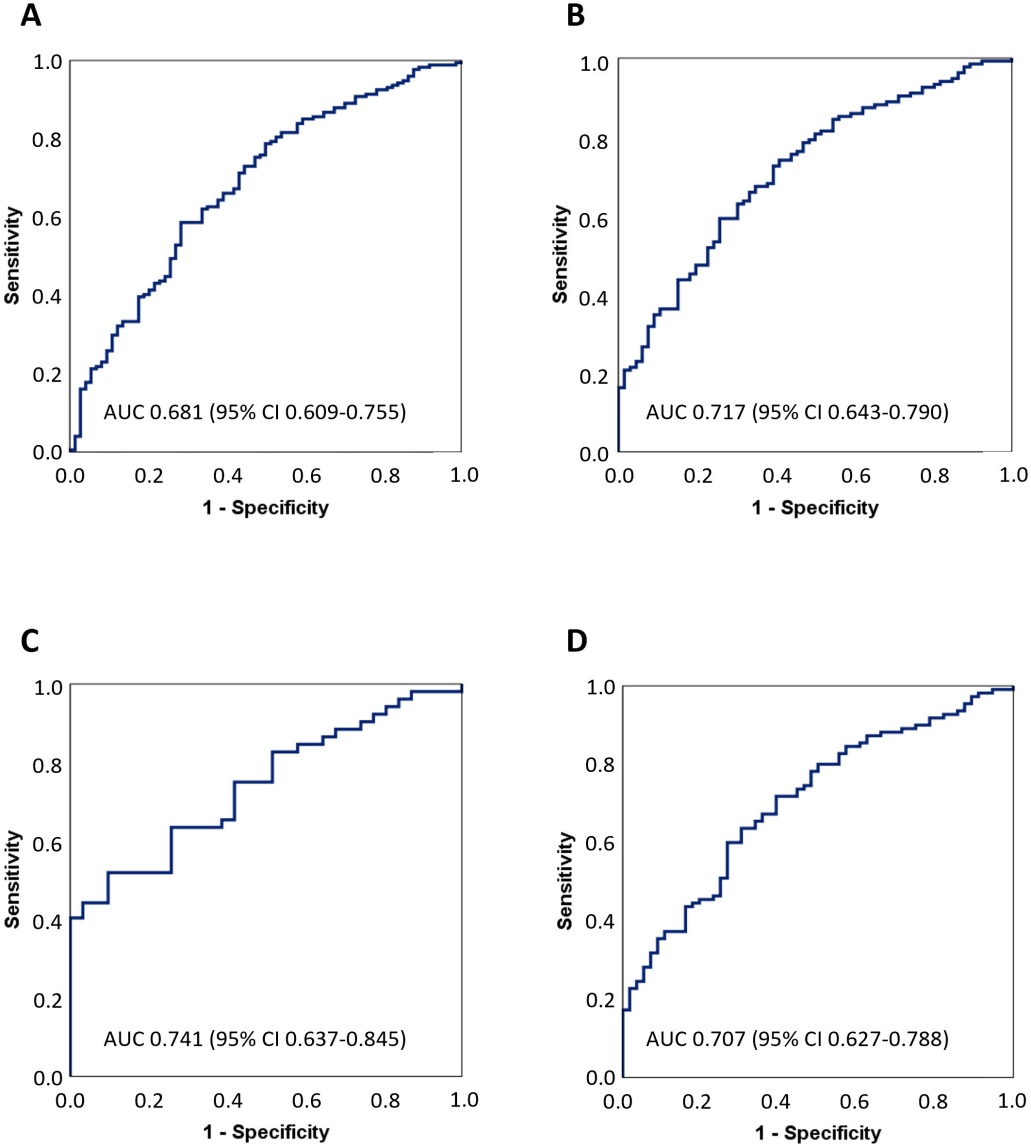
Receiver-operating characteristic curves for differentiating ischemic stroke from hemorrhage stroke patients. Patients with missing creatine values or levels above reference range were excluded from analyses. Area under the curve (AUC) analyses are shown separately for patients with **(A)** NIHSS ≥ 7, *n* = 248; **(B)** NIHSS > 8, *n* = 201; **(C)** NIHSS > 15, *n* = 83; and **(D)** FAST-ED ≥ 4 on admission, *n* = 166. CI, confidence interval; NIHSS, National Institutes of Health Stroke Scale; FAST-ED, Field Assessment Stroke Triage for Emergency Destination score.

As renal insufficiency raises circulating SDMA plasma levels (5), we wanted to verify that intergroup differences remained statistically significant even after excluding patients with admission creatinine levels above the laboratory reference range (>100 μmol/L for men, >90 μmol/L for women, *n* = 61), or with creatinine values missing (*n* = 2). For patients with normal creatinine levels, the intergroup differences in secondary sample SDMA levels remained highly significant in both analyses: 5.68 (5.32–6.42) for IS vs. 4.93 (4.19–5.34) A.U. for HS in MS analysis (*n* = 85, *p* < 0.001), and 0.78 (0.69–0.93) for IS vs. 0.68 (0.56–0.81) nmol/mL for HS in ELISA analysis (*n* = 248, *p* < 0.001). AUCs for differentiating IS and HS patients in ELISA analysis are in Figure 3. For all patients (NIHSS ≥ 7), the discriminatory power was poor (AUC 0.681), but for patients with moderate (NIHSS > 8) and severe symptoms (NIHSS > 15), the discriminatory power was improved (AUCs 0.717 and 0.741, respectively).

Univariate analysis for categorical variables and SDMA levels and correlations between continuous variables and SDMA levels for both patient groups are shown in Tables 3, 4, respectively. In the ELISA analysis, including patients with milder symptoms, we no longer found a correlation with admission NIHSS (ρ = 0.068, *p* = 0.372 for IS patients; ρ = −0.118, *p* = 0.315 for HS). In IS patients, higher plasma SDMA levels were associated with non-smokers (*p* = 0.020) and a history of atrial fibrillation (*p* = 0.029). No significant correlation existed in HS patients between hemorrhage volume and SDMA levels (ρ = −0.118, *p* = 0.071).

**Table 3:** Univariate analysis of categorical variables and SDMA levels of patients with normal creatinine levels in ELISA analysis.

	**Diagnosis class**					
	**IS (*****n** =* **174)**			**HS (*****n** =* **74)**		
	***n***	**SDMA concentration, nmol/mL, median (IQR)**	***p*** **-Value**	***n***	**SDMA concentration, nmol/mL, median (IQR)**	***p*** **-Value**
**Variable**						
**Sex**						
Male	91	0.76 (0.69–0.90)	0.309	48	0.70 (0.59–0.83)	0.167
Female	83	0.80 (0.71–0.94)		26	0.67 (0.53–0.75)	
**Hypertension**						
Yes	104	0.78 (0.70–0.93)	0.471	50	0.68 (0.56–0.75)	0.862
No	70	0.78 (0.68–0.90)		24	0.70 (0.55–0.83)	
**Diabetes**						
Yes	25	0.79 (0.72–0.88)	0.663	8	0.62 (0.54–0.74)	0.384
No	149	0.77 (0.69–0.93)		66	0.69 (0.56–0.81)	
**Hyperlipidaemia**						
Yes	69	0.80 (0.71–0.92)	0.425	21	0.73 (0.62–0.76)	0.483
No	105	0.76 (0.69–0.93)		53	0.68 (0.55–0.81)	
**Previous IS**						
Yes	24	0.78 (0.72–0.95)	0.619	5	0.73 (0.65–0.74)	0.818
No	105	0.78 (0.69–0.93)		69	0.68 (0.56–0.81)	
**Atrial fibrillation**						
Yes	38	0.84 (0.74–1.01)	**0.029**	13	0.68 (0.66–0.81)	0.253
No	136	0.77 (0.69–0.89)		61	0.69 (0.55–0.75)	
**Congestive heart failure**						
Yes	13	0.83 (0.75–0.92)	0.246	4	0.78 (0.65–1.19)	0.278
No	161	0.78 (0.69–0.93)		70	0.68 (0.56–0.81)	
**Coronary artery disease**						
Yes	31	0.85 (0.73–0.97)	0.067	6	0.70 (0.67–0.75)	0.513
No	143	0.76 (0.69–0.91)		68	0.68 (0.55–0.81)	
**Previous myocardial infarction**						
Yes	12	0.85 (0.71–1.02)	0.321	2	0.74 (0.62–0.86)	0.644
No	162	0.77 (0.69–0.91)		72	0.68 (0.56–0.80)	
**Current smoking**						
Yes	54	0.76 (0.60–0.84)	**0.020**	19	0.66 (0.58–0.82)	0.625
No	120	0.80 (0.72–0.94)		55	0.69 (0.55–0.81)	

SDMA, symmetric dimethylarginine; IS, ischemic stroke; HS, haemorrhagic stroke; IQR, interquartile range. Bold indicates *p* < 0.05.

**Table 4:** Correlations of continuous variables and SDMA levels of patients with normal creatinine levels in ELISA analysis.

	**Diagnosis class**					
	**IS (*****n** =* **174)**			**HS (*****n** =* **74)**		
	**n**	ρ	***p*** **-Value**	**n**	ρ	***p*** **-Value**
**Variable**						
Age	174	0.194	**0.010**	74	0.124	0.292
NIHSS on admission	174	0.068	0.372	74	−0.118	0.315
Hemorrhage volume	-	-	-	73	−0.213	0.071
Time from LKW to secondary sample (min)	172	0.020	0.792	71	0.072	0.549
Creatinine levels on admission (umol/l)	174	0.161	**0.034**	74	0.271	**0.019**
HbA1c (mmol/mol)	117	0.086	0.354	47	−0.037	0.803
Total cholesterol (mmol/l)	119	−0.031	0.742	47	0.074	0.623
HDL-cholesterol (mmol/l)	119	−0.045	0.627	47	−0.201	0.175
LDL-cholesterol (mmol/l)	119	−0.058	0.530	47	0.146	0.328
Triglycerides (mmol/l)	119	0.051	0.581	50	0.078	0.591

SDMA, symmetric dimethylarginine; IS, ischemic stroke; HS, haemorrhagic stroke; NIHSS, National Institutes of Health Stroke Scale; LKW, last known well; HbA1c, hemoglobin A1c; HDL, high-density lipoprotein; LDL, low-density lipoprotein. Bold indicates *p* < 0.05.

To use plasma SDMA as a diagnostic biomarker in a potential biomarker panel, we determined, in our ELISA analysis, the optimal plasma SDMA cut-off value for detecting IS patients while reliably ruling out HS. With a SDMA cut-off value of 0.92 nmol/mL and with patients with normal creatinine levels included, we ruled out HS with a high specificity of 90.5% (positive predictive value of 86.5%), but with a very modest sensitivity of 25.9% (negative predictive value of 34.2%).

### 3.5 Stroke etiology and outcome

Of all IS patients, final stroke etiology was cardioembolic (CE) for 100 (44.1%), large-artery atherosclerosis for 42 (18.5%), small-vessel disease for 8 (3.5%), and other cause determined for 6 (2.6%). For 71 (31.3%) IS patients, stroke etiology could not be determined after adequate investigation. Compared to IS patients with another stroke etiology, patients with CE stroke had higher plasma SDMA levels: 0.84 (0.73–1.09) vs. 0.79 (0.71–0.91) nmol/mL (*p* = 0.042).

Having found SDMA to correlate with stroke severity in initial MS analysis, we analyzed whether SDMA levels could predict outcome in all IS patients. Poor outcome at 3 months (mRS 4–6) was associated with higher SDMA levels: 0.90 (0.73–1.06) vs. 0.80 (0.72–0.97) nmol/mL for mRS 0–3 (*p* = 0.045).

## 4 Discussion

Our study of the potential role of SDMA as a metabolite biomarker in the early differential diagnosis of acute stroke patients shows SDMA levels to be significantly higher in IS than in HS patients in the early phase of stroke, with differential diagnostic value primarily in patients with severe symptoms. Even after excluding all patients with creatinine levels above the laboratory reference rate due to SDMA's strong correlation with renal function (5), the intergroup difference remained significant. We also found higher plasma SDMA levels in IS patients to be associated with CE stroke etiology and poor outcome at 3 months.

This is, to our knowledge, the first study to compare SDMA plasma levels in the very early phase of IS and of HS. We evaluated SDMA's diagnostic performance in two phases with two complimentary measuring techniques. First, we used a screening sample of 100 cases with IS or HS for targeted SDMA measurement by mass spectrometry. A further ELISA analysis of a larger sample set of 311 patients then allowed validation of SDMA's diagnostic performance.

Our cohort consists of uniquely early stroke samples with a median delay from LKW to sampling of only 43 min for early and 83 min for secondary samples in the MS analysis, and 89 min for secondary samples in the ELISA analysis. Though no significant differences emerged in our very early prehospital samples, the timepoint of our secondary samples is highly appropriate in the light of other thrombectomy studies (25), with our results highlighting the diagnostic value of SDMA within the first few hours after stroke.

Dimethylarginines, symmetric, and asymmetric dimethylarginine (ADMA), are produced in all nucleated cells when post-translational methylation of arginine residues in proteins is followed by proteolysis (26). ADMA is an inhibitor of NO synthesis, whereas SDMA reduces NO levels indirectly by competing with cellular L-arginine uptake (27). Wide investigation of the role of ADMA has shown it to be associated in various patient populations with cardiovascular risk factors, cardiovascular events, and even death (28). The role of its structural isomer, SDMA, has attracted less study. Recent reports have shown, across various populations, an independent link between increased SDMA concentrations and cardiovascular disease, and all-cause mortality (13). Consistent with the present results, Molnar et al. (6) has suggested that within 6 h of symptom onset, circulating levels of SDMA rise. For now, the mechanism behind this is unclear.

Our finding of significantly higher circulating SDMA levels in IS compared to those in HS patients is in line with earlier findings of elevated SDMA levels in the acute phase of IS compared to levels in patients with asymptomatic significant carotid stenosis and healthy controls (6). Additionally, levels have not been found to significantly increase in the acute phase of HS (8, 9). Among all IS patients in our ELISA analysis, when compared to other stroke etiologies, elevated SDMA levels were most associated with CE stroke (*p* = 0.042). This proved consistent with the findings of Tiedt et al. and Wanby et al. (7, 8). Notably, SDMA levels have been reported to be a potential marker of atrial fibrillation in ESUS stroke patients (29), and in our study, we report an association of atrial fibrillation and higher SDMA levels in IS patients (*p* = 0.029). Our work also supports the notion of higher SDMA levels as being associated in IS patients with poor outcome, as others also suggest (9, 10).

Promising diagnostic biomarkers differentiating IS and HS include GFAP (30, 31), RBP4 (32), PARK7 (33), and apolipoproteins ApoC-I and ApoC-III (34). However, no individual biomarker has yet made it into clinical practice, and accurate out-of-hospital differential diagnosis of stroke patients may require a panel consisting of several independently established complementary biomarkers. Our results indicate that SDMA has value for the development of such diagnostic biomarker panels and is notable as a circulating biomarker that is elevated early after IS, a rare type of finding. Importantly, the best, though only fair, diagnostic value was seen for stroke code patients with severe symptoms (AUC 0.741 for patients with NIHSS > 15; AUC 0.717 for patients with NIHSS > 8; AUC 0.681 for patients with NIHSS ≥ 7), a significant subgroup for LVO diagnostics.

Our study has limitations. To serve as an explorative analysis to best uncover intergroup differences, we included only IS and HS patients with moderate to severe symptoms (NIHSS ≥ 9 for MS analysis and NIHSS ≥ 7 for ELISA). However, patients with suspected acute stroke include those with minor stroke, TIA and stroke mimics. Studies should thus include all patient groups with differing symptom severities. Interestingly, a recent study utilizing untargeted metabolite screening found SDMA to be a promising biomarker for differentiating IS from other conditions mimicking stroke symptoms (7). Secondly, our SDMA values come from two complimentary analyses. As we could not verify the absolute level of quantitation in the MS analysis, we report MS results as arbitrary units. Levels of our ELISA results are consistent with the reference intervals of 0.32–0.65 and 0.225–0.533 nmol/mL reported by El-Khoury et al. and Schwedhelm et al. (35, 36). Importantly, our comparisons were only within these measurement sets, not between them. Thirdly, as SDMA had only fair discriminatory power even for patients with severe symptoms and normal creatinine levels (AUC 0.741), it should be used as a part of a stroke biomarker panel. Furthermore, the strong correlation between SDMA and glomerular filtration rate might limit clinical use of SDMA. However, creatinine point-of-care testing can identify patients with normal kidney function even in a prehospital setting and could be utilized as a part of a biomarker panel to rule out cases with renal insufficiency. Finally, as all EMS units of our district participated in the study recruitment, it was not feasible to achieve consecutive recruitment of all SC patients.

## 5 Conclusions

Our data demonstrate that circulating levels of plasma SDMA elevate in the acute phase of severe IS and can assist in differentiation of early blood biochemical profiles between those in IS and HS during a clinically meaningful acute rime window. Our findings may thus be beneficial in development of stroke biomarker panels for detection of severe stroke when time from onset to sampling is approximately 90 min.

Importantly, our findings concerning SDMA are highly relevant in stroke biomarker discovery, since biomarker elevations have generally appeared in HS where tissue disruption and cellular constituent liberation are sudden, but appear less often in ischemia, where metabolism and biomarker release are typically downregulated before tissue disruption.

## Data Availability

The raw data supporting the conclusions of this article will be made available by the authors, without undue reservation.

## References

[1] Li G Lin Y Yang J Anderson CS Chen C Liu F. Intensive ambulance-delivered blood-pressure reduction in hyperacute stroke. N Engl J Med. (2024) 390:1862–72. 10.1056/NEJMoa2314741 38752650

[2] Pérez de la Ossa N Carrera D Gorchs M Querol M Millán M Gomis M. Design and validation of a prehospital stroke scale to predict large arterial occlusion: the rapid arterial occlusion evaluation scale. Stroke. (2014) 45:87–91. 10.1161/STROKEAHA.113.003071 24281224

[3] Wei H Mundade R Lange KC Lu T. Protein arginine methylation of non-histone proteins and its role in diseases. Cell Cycle. (2014) 13:32–41. 10.4161/cc.27353 24296620 PMC3925732

[4] Nakajima T Matsuoka Y Kakimoto Y. Isolation and identification of N-G-monomethyl, N-G, N-G-dimethyl- and N-G,N' G-dimethylarginine from the hydrolysate of proteins of bovine brain. Biochim Biophys Acta. (1971) 230:212–22. 10.1016/0304-4165(71)90206-6 5573356

[5] Kielstein JT Salpeter SR Bode-Boeger SM Cooke JP Fliser D. Symmetric dimethylarginine (SDMA) as endogenous marker of renal function–a meta-analysis. Nephrol Dial Transplant. (2006) 21:2446–51. 10.1093/ndt/gfl292 16766542

[6] Molnar T Pusch G Papp V Feher G Szapary L Biri B. The L-arginine pathway in acute ischemic stroke and severe carotid stenosis: temporal profiles and association with biomarkers and outcome. J Stroke Cerebrovasc Dis. (2014) 23:2206–14. 10.1016/j.jstrokecerebrovasdis.2014.05.002 25018114

[7] Tiedt S Brandmaier S Kollmeier H Duering M Artati A Adamski J. Circulating metabolites differentiate acute ischemic stroke from stroke mimics. Ann Neurol. (2020) 88:736–46. 10.1002/ana.25859 32748431

[8] Wanby P Teerlink T Brudin L Brattström L Nilsson I Palmqvist P. Asymmetric dimethylarginine (ADMA) as a risk marker for stroke and TIA in a Swedish population. Atherosclerosis. (2006) 185:271–7. 10.1016/j.atherosclerosis.2005.06.033 16055131

[9] Worthmann H Li N Martens-Lobenhoffer J Dirks M Schuppner R Lichtinghagen R. Dimethylarginines in patients with intracerebral hemorrhage: association with outcome, hematoma enlargement, and edema. J Neuroinflammation. (2017) 14:247. 10.1186/s12974-017-1016-1 29237474 PMC5729507

[10] Lüneburg N von Holten RA Töpper RF Schwedhelm E Maas R Böger RH. Symmetric dimethylarginine is a marker of detrimental outcome in the acute phase after ischaemic stroke: role of renal function. Clin Sci. (2012) 122:105–11. 10.1042/CS20110013 21777201

[11] Worthmann H Chen S Martens-Lobenhoffer J Li N Deb M Tryc AB. High plasma dimethylarginine levels are associated with adverse clinical outcome after stroke. J Atheroscler Thromb. (2011) 18:753–61. 10.5551/jat.8144 21566344

[12] Schulze F Carter AM Schwedhelm E Ajjan R Maas R von Holten RA. Symmetric dimethylarginine predicts all-cause mortality following ischemic stroke. Atherosclerosis. (2010) 208:518–23. 10.1016/j.atherosclerosis.2009.06.039 19700158

[13] Schlesinger S Sonntag SR Lieb W Maas R. Asymmetric and symmetric dimethylarginine as risk markers for total mortality and cardiovascular outcomes: a systematic review and meta-analysis of prospective studies. PLoS ONE. (2016) 11:e0165811. 10.1371/journal.pone.0165811 27812151 PMC5094762

[14] Dagonnier M Donnan GA Davis SM Dewey HM Howells DW. Acute stroke biomarkers: are we there yet? Front Neurol. (2021) 12:619721. 10.3389/fneur.2021.619721 33633673 PMC7902038

[15] Llombart V García-Berrocoso T Bech-Serra JJ Simats A Bustamante A Giralt D. Characterization of secretomes from a human blood brain barrier endothelial cells in-vitro model after ischemia by stable isotope labeling with aminoacids in cell culture (SILAC). J Proteomics. (2016) 133:100–12. 10.1016/j.jprot.2015.12.011 26718731

[16] Pihlasviita S Mattila OS Ritvonen J Sibolt G Curtze S Strbian D. Diagnosing cerebral ischemia with door-to-thrombolysis times below 20 minutes. Neurology. (2018) 91:e498–508. 10.1212/WNL.0000000000005954 29997196

[17] Mattila OS Harve H Pihlasviita S Ritvonen J Sibolt G Pystynen M. Ultra-acute diagnostics for stroke: Large-scale implementation of prehospital biomarker sampling. Acta Neurol Scand. (2017) 136:17–23. 10.1111/ane.12687 27642014

[18] Morgenstern LB Lisabeth LD Mecozzi AC Smith MA Longwell PJ McFarling DA. A population-based study of acute stroke and TIA diagnosis. Neurology. (2004) 62:895–900. 10.1212/01.WNL.0000115103.49326.5E 15037689

[19] Rangaraju S Haussen D Nogueira RG Nahab F Frankel M. Comparison of 3-month stroke disability and quality of life across modified Rankin scale categories. Interv Neurol. (2017) 6:36–41. 10.1159/000452634 28611832 PMC5465722

[20] Harbison J Hossain O Jenkinson D Davis J Louw SJ Ford GA. Diagnostic accuracy of stroke referrals from primary care, emergency room physicians, and ambulance staff using the face arm speech test. Stroke. (2003) 34:71–6. 10.1161/01.STR.0000044170.46643.5E 12511753

[21] Lima FO Silva GS Furie KL Frankel MR Lev MH Camargo É. Field assessment stroke triage for emergency destination: a simple and accurate prehospital scale to detect large vessel occlusion strokes. Stroke. (2016) 47:1997–2002. 10.1161/STROKEAHA.116.013301 27364531 PMC4961538

[22] Heldner MR Hsieh K Broeg-Morvay A Mordasini P Bühlmann M Jung S. Clinical prediction of large vessel occlusion in anterior circulation stroke: mission impossible? J Neurol. (2016) 263:1633–40. 10.1007/s00415-016-8180-6 27272907

[23] Roman-Garcia P Quiros-Gonzalez I Mottram L Lieben L Sharan K Wangwiwatsin A. Vitamin B1_2_-dependent taurine synthesis regulates growth and bone mass. J Clin Invest. (2014) 124:2988–3002. 10.1172/JCI72606 24911144 PMC4071367

[24] YueJKYuhELKorleyFKWinklerEASunXPufferRC. Association between plasma GFAP concentrations and MRI abnormalities in patients with CT-negative traumatic brain injury in the TRACK-TBI cohort: a prospective multicentre study. Lancet Neurol. (2019) 18:953–61. 10.1016/S1474-4422(19)30282-031451409

[25] Goyal M Menon BK van Zwam WH Dippel DW Mitchell PJ Demchuk AM. Endovascular thrombectomy after large-vessel ischaemic stroke: a meta-analysis of individual patient data from five randomised trials. Lancet. (2016) 387:1723–31. 10.1016/S0140-6736(16)00163-X 26898852

[26] Teerlink T. ADMA metabolism and clearance. Vasc Med. (2005) 10 Suppl 1:S73–81. 10.1177/1358836X0501000111 16444872

[27] Böger RH. Asymmetric dimethylarginine (ADMA): a novel risk marker in cardiovascular medicine and beyond. Ann Med. (2006) 38:126–36. 10.1080/07853890500472151 16581698

[28] Zhou S Zhu Q Li X Chen C Liu J Ye Y. Asymmetric dimethylarginine and all-cause mortality: a systematic review and meta-analysis. Sci Rep. (2017) 7:44692. 10.1038/srep44692 28294182 PMC5353714

[29] Ziegler NL Sieweke JT Biber S Gabriel MM Schuppner R Worthmann H. Markers of endothelial pathology to support detection of atrial fibrillation in embolic stroke of undetermined source. Sci Rep. (2019) 9:19424. 10.1038/s41598-019-55943-9 31857660 PMC6923420

[30] Mattila OS Ashton NJ Blennow K Zetterberg H Harve-Rytsälä H Pihlasviita S. Ultra-early differential diagnosis of acute cerebral ischemia and hemorrhagic stroke by measuring the prehospital release rate of GFAP. Clin Chem. (2021) 67:1361–72. 10.1093/clinchem/hvab128 34383905

[31] Perry LA Lucarelli T Penny-Dimri JC McInnes MD Mondello S Bustamante A. Glial fibrillary acidic protein for the early diagnosis of intracerebral hemorrhage: systematic review and meta-analysis of diagnostic test accuracy. Int J Stroke. (2019) 14:390–9. 10.1177/1747493018806167 30303809

[32] Bustamante A Penalba A Orset C Azurmendi L Llombart V Simats A. Blood biomarkers to differentiate ischemic and hemorrhagic strokes. Neurology. (2021) 96:e1928–e39. 10.1212/WNL.0000000000011742 33674361

[33] Allard L Burkhard PR Lescuyer P Burgess JA Walter N Hochstrasser DF. PARK7 and nucleoside diphosphate kinase A as plasma markers for the early diagnosis of stroke. Clin Chem. (2005) 51:2043–51. 10.1373/clinchem.2005.053942 16141287

[34] Allard L Lescuyer P Burgess J Leung KY Ward M Walter N. ApoC-I and ApoC-III as potential plasmatic markers to distinguish between ischemic and hemorrhagic stroke. Proteomics. (2004) 4:2242–51. 10.1002/pmic.200300809 15274118

[35] El-Khoury JM Bunch DR Reineks E Jackson R Steinle R Wang S. A simple and fast liquid chromatography-tandem mass spectrometry method for measurement of underivatized L-arginine, symmetric dimethylarginine, and asymmetric dimethylarginine and establishment of the reference ranges. Anal Bioanal Chem. (2012) 402:771–9. 10.1007/s00216-011-5462-9 22124751

[36] Schwedhelm E Xanthakis V Maas R Sullivan LM Atzler D Lüneburg N. Plasma symmetric dimethylarginine reference limits from the Framingham offspring cohort. Clin Chem Lab Med. (2011) 49:1907–10. 10.1515/cclm.2011.679 21864208 PMC3235736

